# Nociceptive transmission and modulation via P2X receptors in central pain syndrome

**DOI:** 10.1186/s13041-016-0240-4

**Published:** 2016-05-26

**Authors:** Yung-Hui Kuan, Bai-Chuang Shyu

**Affiliations:** Division of Neuroscience, Institute of Biomedical Sciences, Academia Sinica, Taipei, 11529 Taiwan Republic of China

**Keywords:** Central pain syndrome, Adenosine triphosphate (ATP), P2X receptors

## Abstract

Painful sensations are some of the most frequent complaints of patients who are admitted to local medical clinics. Persistent pain varies according to its causes, often resulting from local tissue damage or inflammation. Central somatosensory pathway lesions that are not adequately relieved can consequently cause central pain syndrome or central neuropathic pain. Research on the molecular mechanisms that underlie this pathogenesis is important for treating such pain. To date, evidence suggests the involvement of ion channels, including adenosine triphosphate (ATP)-gated cation channel P2X receptors, in central nervous system pain transmission and persistent modulation upon and following the occurrence of neuropathic pain. Several P2X receptor subtypes, including P2X2, P2X3, P2X4, and P2X7, have been shown to play diverse roles in the pathogenesis of central pain including the mediation of fast transmission in the peripheral nervous system and modulation of neuronal activity in the central nervous system. This review article highlights the role of the P2X family of ATP receptors in the pathogenesis of central neuropathic pain and pain transmission. We discuss basic research that may be translated to clinical application, suggesting that P2X receptors may be treatment targets for central pain syndrome.

## Background

According to the International Association for the Study of Pain (IASP), central neuropathic pain is commonly caused by lesions or disease of the central somatosensory nervous system (e.g., brain trauma, stroke, multiple sclerosis, spinal cord injury, syringomyelia, syringobulbia, tumors, and abscesses) or other inflammatory central nervous system (CNS) diseases [1]. The National Institute of Neurological Disorders and Stroke (NINDS) notes that central pain syndrome is a neurological condition that is caused by damage to or dysfunction of the CNS, including the brain, brainstem, and spinal cord. This syndrome can be caused by stroke, multiple sclerosis, tumors, epilepsy, brain or spinal cord trauma, or Parkinson’s disease [2].

Because of the wide variety of potential causes, the characteristics of pain that is associated with this syndrome differ widely among individuals. According to the NINDS, central pain syndrome can affect both specific areas, such as the hands or feet, or a large portion of the body. Prolonged pain is usually related to the cause of CNS injury, and the resulting central pain is typically perceived as constant, with moderate to severe intensity, and often made worse by touch, movement, and changes in temperature. Individuals might experience one or more types of pain sensations, the most prominent of which is a burning sensation that is sometimes combined with sensations of pressing, laceration, or aching and brief, intolerable bursts of sharp pain. Such sharp pain is similar to the pain that may be caused by a dental probe that touches an exposed nerve [2]. Individuals may also experience numbness in areas that are affected by this pain syndrome. Some individuals claim that burning feelings and loss-of-touch sensations are usually most severe in distal parts of the body, such as the hands and feet. Central pain syndrome often begins shortly after the causal injury or damage but may be delayed by months or even years, especially if it is related to post-stroke pain [2].

Molecular studies, particularly focused on ion channels that are related to plasticity in the nociceptive somatosensory system, may enable the development of new pharmacological treatments against central pain syndrome. To date, evidence suggests the involvement of various ion channels in the pathogenesis of central pain, including sodium channels, calcium channels, potassium channels, acid-sensing ion channels, and adenosine triphosphate (ATP)-gated cation channels [3–7]. In this review, we focus on the involvement of ATP-gated cation channel P2X receptors in central nervous system pain transmission and persistent modulation upon and following the occurrence of central neuropathic pain. We discuss basic research that may be translated to clinical application, suggesting that P2X receptors may be treatment targets for central pain syndrome.

### Adenosine triphosphate and the involvement of P2X Receptors in neuropathic pain

Adenosine triphosphate (ATP) is a well-known energy provider for cellular function. It was discovered to act as a neurotransmitter in the early 1970s [8]. This opened the way for studies on purinergic signaling and related receptors, thus spurring the field of purinergic neurotransmission [9]. In the CNS, ATP serves as a signaling molecule that modulates communication between neurons and glial cells. Cellular responses to trauma and ischemia that are trigged by ATP are important for initiating and maintaining reactive astrogliosis, which may result in changes in the proliferation and morphology of astrocytes and microglia [10]. A small amount of ATP that is released during brain injury is neuroprotective; at higher concentrations, however, ATP can contribute to pathogenic processes [11]. Channel characterization provided a basis for distinguishing two types of purinoceptors, P1 and P2. P1 receptors are specific to nucleosides, and P2 receptors are specific to nucleotides [12]. P1 receptors are G protein-coupled receptors, and P2 receptors can be divided into P2X ion channel receptors and P2Y G protein-coupled receptors [13].

P2X receptors attracted scientific interest in pain research with early observations that ATP itself evokes pain when applied to blisters [14, 15]. Accumulating evidence indicates that this effect is mediated by the activation of P2X3 receptors, which are restricted in their distribution to a subset of primary afferent neurons [15–17] that also express receptors for capsaicin (TRPV1) and isolectin B4 [15, 18, 19]. P2X3 receptors also play a role in visceral mechanosensory transduction. Distension-induced stimulation of P2X3 receptors that are expressed on primary sensory afferents in the urothelium was shown to result in ATP release, leading to an increase in afferent nerve activity. P2X3 knockout mice and animals with alterations in P2X3 levels exhibited a decrease in afferent nerve activity and a reduction of nociceptive signaling [20, 21]. Previous reviews suggested that P2X3 receptors and the P2X2*/*3 receptor complex play crucial roles in mechanosensory transduction and nociception [22]. Antagonists that are selective for P2X3-containing receptors have been shown to reduce chronic neuropathic and inflammatory pain in rats [23]. The downregulation of P2X3 receptor expression by intrathecal antisense oligonucleotide administration or local administration of the P2X2/3 antagonist A-317491 reduced mechanical hyperalgesia that was evoked by carrageenan [24] and complete Freund’s adjuvant [25]. These studies demonstrated that ATP acts on P2X2/3 receptors that are expressed on peripheral nerve afferents as a component of the local inflammatory response.

Models of neuropathic pain have revealed that both locally and intrathecally applied selective P2X3 receptor antagonists can prevent mechanical allodynia [26, 27]. When applied locally, P2X3 antagonists cause sensitization and an increase in the expression of membrane P2X3 receptors, rather than an increase in ATP release [28].

P2X4 receptors that are expressed in the CNS have been proposed to be involved in the pathogenesis of neuropathic pain [29]. The development of oversensitivity to mechanical stimuli following peripheral nerve injury was prevented by P2X4 receptor depletion, and allodynia that occurs post-nerve injury may be reduced by targeting P2X4 receptors [30–32]. The activation of spinal microglia is known to occur following peripheral nerve injury. Evidence suggests that ATP acts on P2X4 receptors to drive the release of brain-derived neurotrophic factor (BDNF) from spinal microglia, which is a critical response for neuronal tissue rewiring that underlies the perception of mild tactile stimuli as noxious [32].

P2X7 receptors have been shown to modulate behavioral responses to painful stimuli [33, 34]. In contrast to P2X4 receptors, pharmacological antagonists of P2X7 receptors are available as experimental tools, and some of these have been tested in clinical trials [26, 35]. P2X7 receptors are predominantly expressed on microglia, astrocytes, and oligodendrocytes in the nervous system. However, some reinterpretation of the data from experiments that used P2X7 knockout mice is required. P2X7 knockout mice that are referred to as the Pfizer line (generated by Solle and colleagues [36]) continue to express two alternatively spliced and shortened forms of P2X7 receptors (i.e., P2X7 13B and P2X7 13C). P2X7 knockout mice that are provided by Glaxo continue to express a functional P2X7 splice variant (P2X7k), and the corresponding protein is widely expressed but has a different N-terminus and transmembrane domain 1 (TM1) [37]. The P2X7k splice variant forms receptors that are more sensitive to ATP and undergo a rapid increase in permeability to organic cations, a measure of pore dilation [37].

In models of neuropathic pain, the development of mechanical hypersensitivity is absent in Glaxo P2X7 knockout mice [38]. A similar phenotype is observed in mice that lack both isoforms of interleukin-1β (IL-1β) [39]. Intrathecal administration of the P2X7 antagonist A-438079 may prevent mechanical hypersensitivity [40], and the same phenomenon was observed with systemic administration of Brilliant Blue G (BBG) [41] and systemic administration of other P2X7 receptor antagonists [42]. These effects in models of pain appear to require the release of IL-1β. A-438079 was shown to block both the ATP- and lipopolysaccharide (LPS)-induced release of IL-1β [43]. Indeed, the LPS-induced release of IL-1β is absent in lumbar spinal cord slices from P2X7 knockout mice. These results suggest that the rewiring of microcircuitry in the spinal cord that occurs during prolonged inflammation or nerve injury may require the involvement of sequential signaling that is initiated with ATP release, followed by the activation of P2X7 receptors on spinal microglia and release of IL-1β. Our understanding of the source of ATP has not advanced much since Pamela Holton showed that sensory nerves release ATP in response to electrical stimulation [44]. IL-1β release that is caused by nerve injury or prolonged inflammation has been suggested to lead to an increase in the phosphorylation of the NR1 and NR2B subunits of the *N*-methyl-D-aspartate receptor and perhaps an LTP-like phenomenon [45]. Such responses have been associated with behavioral hyperalgesia [46].

P2X7 receptors have been considered a potential target for pain therapies, which is supported by genetic association studies. The function of a particular form of mouse P2X7(P451L) receptors is impaired when measured by the ATP-evoked uptake of YO-PRO-1 (i.e., a commonly used optical measure of pore dilation) but not when measured by ATP-induced ionic currents [47, 48]. The H155Y and R270H polymorphisms of P2X7 receptors are known to result in an increase and decrease in P2X7 receptor function, respectively. The H155Y and R270H polymorphisms of P2X7 were shown to be expressed more often in subjects who reported higher and lower levels of pain, respectively, following mastectomy [49]. These authors utilized seven inbred strains, each with different P2X7 alleles that resulted in various single-nucleotide polymorphisms (SNP). Six strains that carried the Leu451 allele presented significantly lower allodynia compared with strains that carried the Pro451 allele [49].

### P2X receptors and neuropathic pain caused by spinal cord injury

Adenosine triphosphate has been shown to be a key element in relaying sensory information from the periphery to the CNS [50], in addition to being one of several important mediators of immune-neural interactions [51]. Both sensory neurons and glial cells inside and outside the CNS release ATP to affect surrounding cells [52, 53]. Accumulating evidence suggests a link between activated microglia and astrocytes, central sensitization, and the development and maintenance of neuropathic pain [53–55]. Spinal cord injury is often complicated by secondary injury as a result of the innate inflammatory response to tissue trauma and swelling. In the setting of spinal cord injury, ATP release increases in peritraumatic areas for > 6 h [56]. Below we discuss studies of the involvement of P2X receptors in neuropathic pain that is caused by spinal cord injury.

P2X3 receptors were shown to be expressed on rat sensory neurons in naive animals, and immunohistochemistry revealed elevations of P2X3 receptor expression in the superficial laminae of the dorsal horn following nerve injury [57]. A significant increase in the number of nodose ganglion neurons that expressed P2X3 was found upon spinal cord injury, a finding that matched also for the bladder-innervating vagal afferents, because most viscera, including the bladder, are dually innervated by spinal and vagal sensory neurons and the impacts of spinal cord injury on the sensory component of vagal circuitry was shown to have a significant increase in the number of NG neurons expressing P2X3 and this may contribute to visceral pathologies following spinal cord injury [58]. Munoz and colleagues found that changes in bladder afferent signals in normal and spinal cord-injured rats may be modulated by purinergic P2X3 and P2X2/3 receptors [59]. These studies indicate that vagal afferents, including those that innervate the bladder, exhibit neurochemical plasticity after spinal cord injury, which may elucidate the visceral homeostatic mechanisms and nociceptive signaling that are related to the involvement of P2X3 and P2X2/3 receptors. A novel non-nucleotide antagonist of P2X3 receptors and the P2X2/3 receptor complex was shown to block native P2X3 and P2X2/3 receptors on rat dorsal root ganglion neurons and attenuate both thermal hyperalgesia and mechanical allodynia after chronic spinal cord injury [23].

Tsuda and colleagues reported that P2X4 receptors are expressed in neurons in the spinal cord [30]. Later reports provided direct evidence that spinal cord injury causes an innate inflammatory response that leads to an increase in caspase-l cleavage and the production of IL-1β [32, 50, 51, 60]. P2X4 knockout mice exhibited impairments in inflammasome signaling in the spinal cord, resulting in a decrease in the levels of IL-1β, reductions of the infiltration of neutrophils and monocyte-derived M1 macrophages, significant tissue sparing, and improvements in functional outcomes [29, 31, 32]. These results indicate that P2X4 receptors influence inflammasome signaling, caspase-1 activation, and IL-1β processing in neurons after spinal cord injury. Thus, P2X4 receptors might be a potential therapeutic target to limit inflammatory responses that are associated with spinal cord injury and neurodegenerative disorders [29, 60].

P2X7 receptors were shown to be elevated after spinal cord injury as well, inhibition of this elevation of P2X7 resulted in improvements in the recovery from spinal cord injury in a rat model [56]. P2X7 receptor activation was observed in a weight-drop model of thoracic spinal cord injury in rats, and systemic administration of the P2X7 receptor antagonist BBG exerted neuroprotective effects. The administration of BBG within 15 min after injury reduced tissue damage in the spinal cord and improved motor recovery without causing overt cellular/tissue toxicity [61].

These reports indicate that P2Xs are expressed on sensory neurons in the spinal cord and are activated after various forms of spinal cord injury, and subsequent ATP signaling is involved in the pathogenesis of central neuropathic pain.

### P2X receptors and central post-stroke pain

Central post-stroke pain (CPSP) is defined by the IASP as a central neuropathic pain condition, in which pain arises as a direct result of cerebrovascular lesions in the central somatosensory nervous system [2]. To date, few reports have investigated the involvement of P2X receptors in CPSP. Some studies have reported that peripheral ATP and purinergic P2X receptors on sensory nerve terminals contribute to spinal cord sensitization, which may be involved in CPSP-related behavior [62]. Microglia express numerous purinergic receptors. P2X4 and P2X7 receptors have been shown to play important roles in central neuropathic pain via microglia activation [63–65]. The inhibition of P2X4 receptors after nerve injury has been shown to prevent the upregulation of P2X4 receptors in the dorsal horn, alleviate mechanical allodynia [66], and influence the progression of traumatic injury in the CNS [67, 68]. The genetic elimination of P2X7 receptors resulted in a decrease in neuropathic pain, but unclear is whether this effect is specifically mediated by microglia, particular subtypes of central neurons within specific microcircuitry, or peripheral cell types [38, 69].

### Functional involvement of P2X7 receptors in CPSP

A recent study by our group investigated the involvement of P2X7 receptors in a rat model of CPSP and the mechanisms that are involved in the activation of P2X7 receptor pathways in the pathogenesis of CPSP. A thalamic hemorrhagic rat model was used, in which type IV collagenase was injected into the right lateral thalamus. Behavioral tests, electrophysiological recordings, and microdialysis were performed to elucidate the characteristics of CPSP. Systemic and local pharmacological interventions were used to interrupt P2X7 receptor activation. Spontaneous pain and thermal and mechanical hyperalgesia developed in the subacute to chronic phases in rats that were subjected to lateral thalamic hemorrhage. Immunostaining at 5 weeks post-hemorrhage revealed significant activation of P2X7 receptors in microglia along thalamic peri-lesion tissue. Increases in the immunoreactivity of P2X7 receptors and the reactive microglia marker CD11b were largely localized to peri-lesion sites in CPSP rats, whereas their immunoreactivity remained at basal levels in the contralateral site and sham control rat brains. Significant increases in the counts of cells that were immunoreactive to both P2X7 and CD11b in a selected region of interest were observed. Tissue samples from the peri-lesion sites were screened using quantitative reverse-transcription polymerase chain reaction. The levels of tumor necrosis factor α (TNF-α), IL-6, and BDNF significantly increased in peri-lesion tissues compared with unaffected contralateral sites. The proinflammatory cytokine IL-1β directly downstream of P2X7 receptor activation also exhibited a prominent elevation.

Thalamocingulate circuitry in the CNS is known as a medial pain processing pathway. Changes in nociceptive sensitivity in the forebrain under conditions of neurogenic pain likely result from aberrant neuronal activity along this pathway, reflected by thalamocortical dysrhythmia [70–72]. To test the hypothesis that persistent pain in CPSP rats is accompanied by changes in nociceptive sensitivity, multichannel electrodes were used to record evoked neuronal activity in the anterior cingulate cortex (ACC) and medial thalamus (MT) in response to sciatic nerve stimulation. Treatment with a P2X7 receptor antagonist during the acute stage of hemorrhage rescued abnormal pain behaviors and neuronal activity in the thalamocingulate pathway by reducing microglial aggregation and associated inflammatory cytokines. Aberrant spontaneous thalamocortical oscillations after lateral thalamic hemorrhage could be regulated by blocking P2X7 receptors. These findings suggest that P2X7 receptors may be a potential target for preventing the occurrence of CPSP in patients with acute stroke [73].

**Fig. 1:**
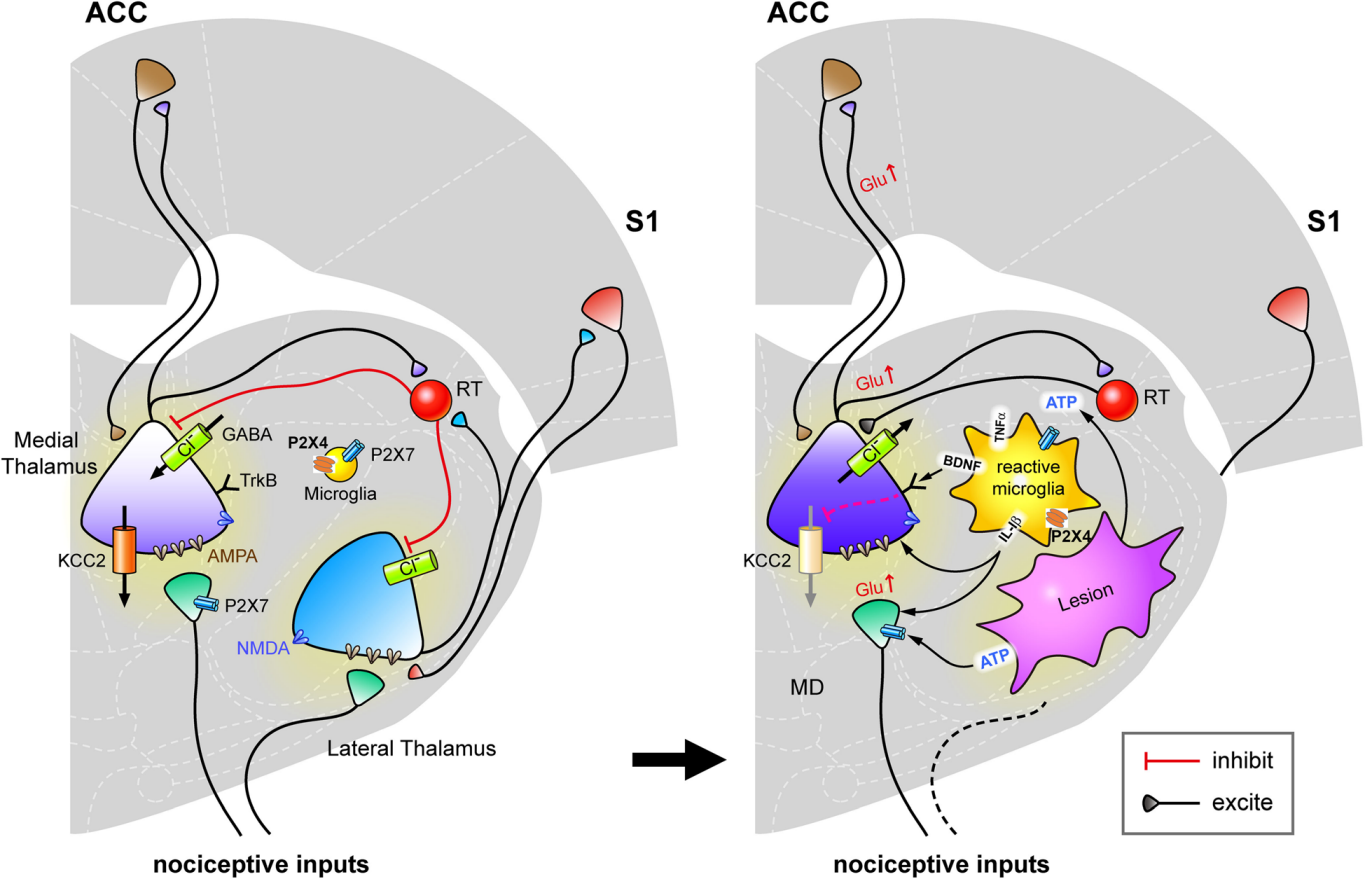
Schematic illustration of P2X7 receptor involvement in sequential signaling effects on microglia and neuronal activity in CPSP. The figure illustrates our concepts of the involvement of P2X7 receptor in normal and CPSP states, combined with previous reports of P2X4 and P2X7 receptor function and their roles in other types of neuropathic pain. In the normal state, P2X4 and P2X7 receptor expression is low in neurons, astrocytes, glia, and resting microglial cells. In the CPSP state, the pathological enhancement of ATP release to surrounding tissue after stroke insult may lead to an increase in both P2X4 and P2X7 receptor expression and activation. Such activation has been reported to promote synaptic glutamate release and the abnormally high production of IL-1β, TNF-α, and BDNF. Consequently, IL-1β has also been reported to alter glutamate transmission in the CNS, and BDNF has been shown to alter chloride ion flux via TrkB and GABA receptors. Such sequential elevations of signaling molecules at sites of damage, combined with blood cell infiltration, can affect the activity of astrocytes, microglia, and neurons, thus resulting in neuronal hyperexcitability that is promoted by peripheral pain sensitivity and leading to the pathogenesis of CPSP. Figure adapted from Kuan et al. [68]

Figure 1 illustrates our concepts regarding P2X7 receptor in normal and CPSP states, combined with previous reports of P2X4 and P2X7 receptor function and their role in other types of neuropathic pain. Both of these receptor subtypes have been shown to be highly involved in neuropathic inflammatory pain [29–35]. In the normal state, P2X4 and P2X7 receptor expression is low in neurons, astrocytes, glia cells, and resting microglia cells. In CPSP, however, the pathological enhancement of ATP release to surrounding tissue after stroke insult may lead to an increase in both P2X4 and P2X7 receptor expression and activation. Such activation has been reported to promote synaptic glutamate release and the abnormally high production of IL-1β [74–76], TNF-α, and BDNF [24–27]. Consequently, IL-1β has been reported to alter glutamate transmission in the CNS [77], and BDNF has been shown to alter chloride ion flux via TrkB and γ-aminobutyric acid receptors [78]. Such sequential elevations of signaling molecules at sites of damage, combined with blood cell infiltration, can affect the activity of astrocytes, microglia, and neurons, thus resulting in neuronal hyperexcitability that is promoted peripheral pain sensitivity and contributing to the pathogenesis of CPSP.

### Prospective therapeutic implications

The P2X family of ATP receptors is widely expressed in various tissues. P2X2, P2X3, P2X4, and P2X7 receptors are more abundant in central and peripheral neurons [79–81]. Under pathological conditions, ATP is the most powerful mediator of P2X receptor activation. P2X receptors, including neuronal P2X3 receptor subtypes and P2X4 and P2X7 receptors that are expressed on non-neuronal cells, behave as sensitive ATP detectors. Dissecting the molecular mechanisms in sensory neurons and accessory cells may contribute to the development of tissue- and cell-targeted approaches to treat chronic pain and inflammatory diseases [82]. P2X3, P2X4, and P2X7 receptor antagonists have been shown to have antinociceptive and antiinflammatory effects in animal models of these diseases [38, 56, 62, 63, 65, 83, 84]. As illustrated in Fig. 1, a logical proposal is that targeting P2X7 receptors after the onset of CPSP may block the hyperexcitability that is observed in thalamocingulate pathways and block the abnormal elevation of P2X7 receptor activation, culminating in the prevention of abnormal glutamate release and IL-1β secretion and modulation of neuronal activity and behavioral pain under conditions of CPSP.

Central pain syndrome is a substantial clinical problem, but the currently available treatments are unable to provide sufficient pain relief for patients who suffer from the chronic phase of this condition. Previous studies by our group suggest the possibility of targeting P2X7 receptors during the initial onset of stroke or delaying treatment until CPSP is manifest in the subacute to chronic phase. In stroke patients who already suffer from CPSP, agents that target P2X7 receptors may exert antinociceptive effects by suppressing or blocking neuronal hyperexcitability and reversing abnormal oscillations. Early treatment with a P2X7 receptor antagonist in stroke patients may prevent the activation of P2X7 receptors that are expressed on microglia/monocyte-derived macrophages in peri-lesion tissues, thus reducing the release of regional inflammatory cytokines and associated neuronal damage. Importantly, commercially available P2X7 receptor antagonists are usually small-molecule compounds with good blood–brain barrier permeability when administered peripherally. We found that systemic infusion of BBG did not cause apparent toxicity, which confirms previous findings [61] and suggests that similar compounds that target various P2X receptors may be therapeutic candidates for the treatment of central pain syndromes that are caused by brain trauma.

## Conclusion

The overall complexity of the plasticity of pain receptors, the sensitization of sensory neurons, and mediators that are released from non-neuronal cells has hindered our understanding of the spatial and temporal development of pain, neuronal responses, and disease progression. We have primarily focused on the involvement of P2X receptors in central neuropathic pain that is caused by nerve injury. Sites of the expression of these P2X receptors and pharmacological, molecular, and genetic manipulations of their function have reveled clear influences on nerve injury-induced pain behaviors and hyperexcitability of the central pain pathway. P2X3 receptors play a significant role in neuropathic and inflammatory pain. Long-lasting allodynia that is produced by intrathecal administration of ATP likely occurs through P2X2/3 receptors. Spinal P2X2 and P2X3 receptors have been reported to be involve in neuropathic pain in a mouse model of chronic constriction injury. Dorsal horn neurons relay nociceptive information along the pain pathway via P2X4 receptors. P2X receptor activation in the spinal cord may elicit allodynia, and the upregulation of P2X4 receptors in spinal cord microglia appears to play a dominant role in this effect. P2X7 receptors, via the regulation of IL-1β production, play an upstream transductional role in the development of neuropathic and inflammatory pain. Reviews of the role of P2X7 receptors in pain and inflammation highlight the potential therapeutic benefit of modulating P2X7 receptors. However, the underlying mechanisms that involve microglial P2X4 and P2X7 receptors are still unclear. The present review highlights the involvement of the P2X family of ATP receptors in the pathogenesis of central neuropathic pain and overall pain transmission. A better understanding of the function and involvement of P2X receptors will provide useful insights into mechanisms of pain and possibly new therapeutic agents for the management of neuropathic pain. P2X receptors should be considered further as treatment targets for central pain syndrome.

**Fig. 2:**
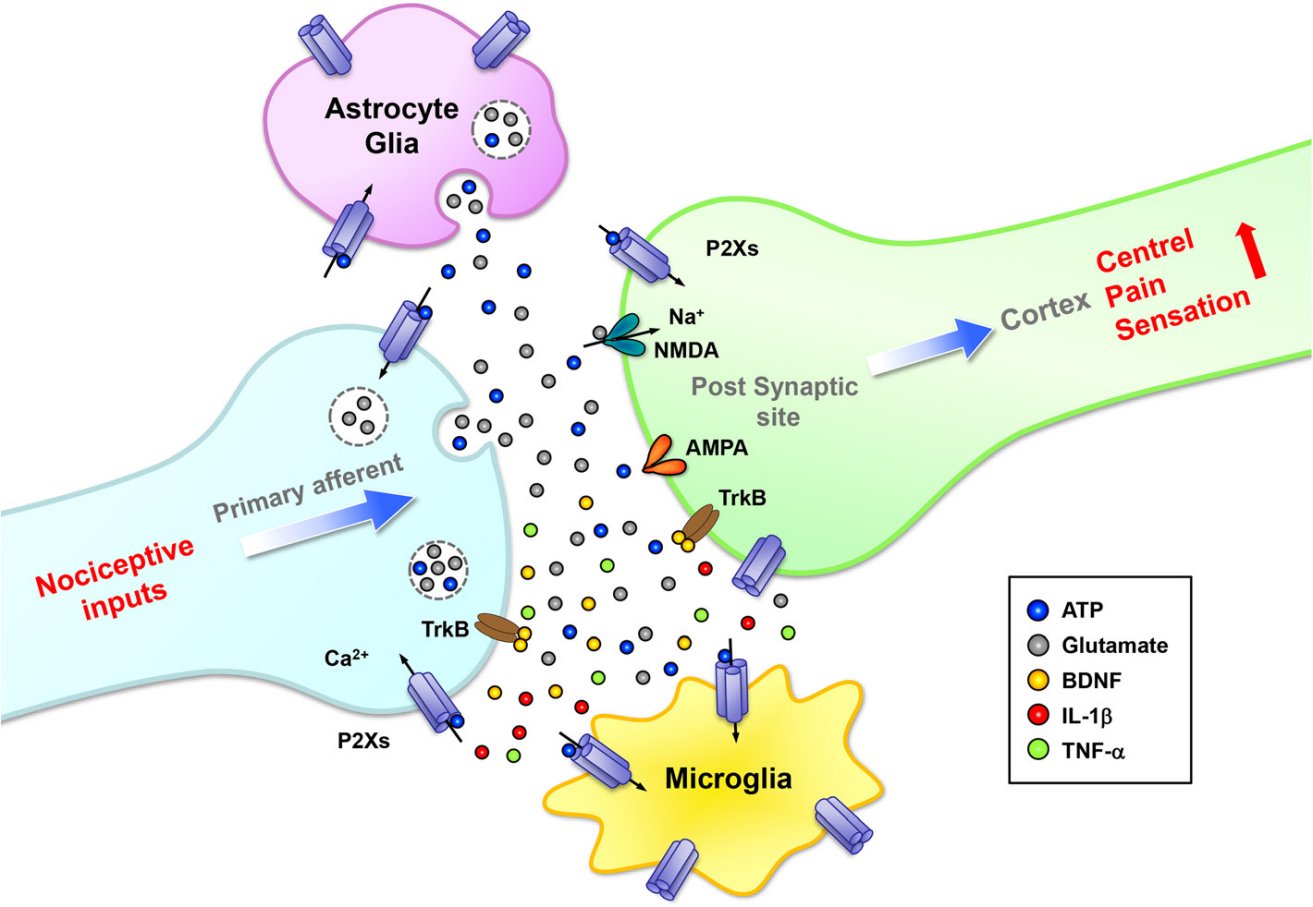
Schematic illustration of P2X receptors involvement in nociceptive transmission and modulation in central pain syndrome. Adenosine triphosphate is an important soluble mediator that is involved in cross-talk between sensory neuron synapses within ascending nociceptive transmission pathways. P2X receptors are expressed in the presynaptic membrane, postsynaptic density, astrocytes, and microglia. In the central pain state, abnormal neuronal excitability and enhanced glutamate/ATP release activate postsynaptic neurons, microglia, and astrocytes, thus contributing to central sensitization and the release of inflammatory mediators and neurotrophins. Currently available evidence strongly suggests a key modulatory role of P2X receptors in central sensitization. Primary nociceptive inputs promote glutamate and ATP co-release and synergistically cause non-selective permeability to Ca^2+^, Na^+^, and K^+^ ions via P2X receptors, leading to the postsynaptic activation of NMDA or AMPA receptors and further contributing astrocytic glutamate and ATP co-release into the extracellular milieu. Intracellular astrocytic Ca^2+^ signals may spread within astrocytes, leading to similar effects at other synapses. Local brain tissue damage resulted ATP release and inflammation can also activate extrasynaptic P2X receptors on microglia. P2X receptors expressed on astrocytes and microglia induces a local inflammatory response with release of cytokines. P2X7 has many consequences in pain, for its role in the inflammasome activation and maturation of IL-1β, one of the most powerful mediators of acute inflammatory response. P2X4 was reported to induce release of BDNF, acting on TrkB receptor which modulates inhibitory neurons, contributing to exacerbation of painful signal transmission pathways in brain centers. Both synaptic and glial release of glutamate and the co-release of neuronal, astrocytic and microglial ATP contribute to synaptic transmission via P2X receptors

Figure 2 illustrates our concepts of the involvement of P2X receptors in nociceptive transmission and modulation in central pain syndrome. Adenosine triphosphate is an important soluble mediator that is involved in cross-talk between sensory neuron synapses within ascending nociceptive transmission pathways. P2X receptors have been reported to express in the presynaptic membrane, postsynaptic density, astrocytes, and microglia. In central pain states, abnormal neuronal excitability and enhanced glutamate/ATP release activate postsynaptic neurons, microglia, and astrocytes, thus contributing to central sensitization and the release of inflammatory mediators and neurotrophins. Currently available data strongly suggest a key modulatory role of P2X receptors in central sensitization [82, 85]. Primary nociceptive inputs promote glutamate and ATP co-release and synergistically cause the release of intracellular Ca^2+^ within neuronal or astrocyte processes, leading to the postsynaptic activation of N-methyl-D-aspartate (NMDA) or α-amino-3-hydroxy-5-methyl-4-isoxazolepropionic acid (AMPA) receptors and further astrocytic ATP release into the extracellular milieu. Local brain tissue damage can activate extrasynaptic P2X receptors on microglia. Astrocytic Ca^2+^ signals may spread within astrocytes, leading to similar effects at other synapses. Microglia activation in responses to local inflammatory condition resulting release of cytokines. P2X7 has many consequences in pain, for its role in the inflammasome activation and maturation of IL-1β, one of the most powerful mediators of acute inflammatory response. P2X4 was reported to induce release of BDNF, acting on TrkB receptor which modulates inhibitory neurons, contributing to exacerbation of painful signal transmission pathways in brain centers. Both synaptic and glial glutamate release and the co-release of neuronal, astrocytic and microglial ATP contribute to synaptic transmission. However, astrocytic and microglial processes have not yet been shown to actively contribute to synaptic transmission; instead, they exert strong activation-dependent neuromodulation via ATP. The fine control of P2X receptor activity in different cell types is thus thought to be necessary for maintaining tissue in a healthy functional state.

## Abbreviations

ACC; Anterior cingulate cortex, ATP, Adenosine triphosphate; BBG, Brilliant Blue G; BDNF, Brain-derived neurotrophic factor; CNS, central nervous system; CPSP, Central post-stroke pain; IASP, International Association for the Study of Pain; IL-1β, Interleukin-1β; LPS, Lipopolysaccharide; MT, Medial thalamus; NINDS, National Institute of Neurological Disorders and Stroke; SNP, Single-nucleotide polymorphism; TM1, Transmembrane domain 1; TNF-α, Tumor necrosis factor α.
